# Clinical characteristics and prognosis of acute bacterial meningitis in elderly patients over 65: a hospital-based study

**DOI:** 10.1186/1471-2318-11-91

**Published:** 2011-12-28

**Authors:** Wei-An Lai, Shu-Fang Chen, Nai-Wen Tsai, Chiung-Chih Chang, Wen-Neng Chang, Cheng-Hsien Lu, Yao-Chung Chuang, Chun-Chih Chien, Chi-Ren Huang

**Affiliations:** 1 Department of Family Medicine, Kaohsiung Chang Gung Memorial Hospital and Chang Gung University College of Medicine, Kaohsiung City, Taiwan; 2 Department of Neurology, Kaohsiung Chang Gung Memorial Hospital and Chang Gung University College of Medicine, Kaohsiung City, Taiwan; 3 Department of Biological Science, National Sun Yat-Sen University, Kaohsiung City, Taiwan; 4 Department of Diagnostic Pathology, Kaohsiung Chang Gung Memorial Hospital and Chang Gung University College of Medicine, Kaohsiung City, Taiwan

## Abstract

**Background:**

To examine the clinical characteristics of bacterial meningitis in elderly patients.

**Methods:**

261 patients with adult bacterial meningitis (ABM), collected during a study period of 11 years (2000-2010), were included for study. Among them, 87 patients aged ≥ 65 years and were classified as the elderly group. The clinical and laboratory characteristics and prognostic factors were analyzed, and a clinical comparison with those of non-elderly ABM patients was also made.

**Results:**

The 87 elderly ABM patients were composed of 53 males and 34 females, aged 65-87 years old (median = 71 years). Diabetes mellitus (DM) was the most common underlying condition (34%), followed by end stage renal disease (7%), alcoholism (4%) and malignancies (4%). Fever was the most common clinical manifestation (86%), followed by altered consciousness (62%), leukocytosis (53%), hydrocephalus (38%), seizure (30%), bacteremia (21%) and shock (11%). Thirty-nine of these 87 elderly ABM patients had spontaneous infection, while the other 48 had post-neurosurgical infection. Forty-four patients contracted ABM in a community-acquired state, while the other 43, a nosocomial state. The therapeutic results of the 87 elderly ABM patients were that 34 patients expired and 53 patients survived. The comparative results of the clinical and laboratory characteristics between the elderly and non-elderly ABM patients showed that only peripheral blood leukocytosis was significant. Presence of shock and seizure were significant prognostic factors of elderly ABM patients.

**Conclusions:**

Elderly ABM patients accounted for 34.8% of the overall ABM cases, and this relatively high incidence rate may signify the future burden of ABM in the elderly population in Taiwan. The relative frequency of implicated pathogens of elderly ABM is similar to that of non-elderly ABM. Compared with non-elderly patients, the elderly ABM patients have a significantly lower incidence of peripheral blood leukocytosis. The mortality rate of elderly ABM remains high, and the presence of shock and seizures are important prognostic factors.

## Background

Elderly people are vulnerable to infectious diseases, and may present with fewer classic signs and symptoms for clinical identification [1]. The study of bacterial meningitis with a focus only on elderly patients has rarely been solely examined in the related literature [2-4]. In the United States, because of the success of conjugate vaccines in reducing the risk of major pathogens-related meningitis among young children, the burden of bacterial meningitis is now borne more by older patients [5]. It is known that several factors including age, geographic distribution, underlying medical and/or surgical conditions, mode of contraction, the study time period, and the status of vaccination may influence the prevalence of causative pathogens of bacterial meningitis [1,6,7]. Furthermore, the epidemiologic change of implicated pathogens may influence the early choice of appropriate, empiric antibiotics which is important for the successful treatment of adult bacterial meningitis (ABM) [1,6,7]. Elderly patients with bacterial meningitis are known to have a grave prognosis [3,4]. Therefore, in this study, we analyzed the clinical and laboratory characteristics and the therapeutic outcome of 87 elderly ABM patients and also made a clinical comparison with those of with bacterial meningitis in a non-elderly group.

## Methods

We retrospectively reviewed microbiological records for cerebrospinal fluid (CSF) and the medical records of patients with ABM admitted to the Chang Gung Memorial Hospital (CGMH)-Kaohsiung over a period of 11 years (2000 - 2010). CGMH-Kaohsiung is the largest medical center in southern Taiwan and the facility is a 2,482-bed acute-care teaching hospital, which serves as a primary and tertiary care center. In this study, the criteria for a definite diagnosis of ABM were as follows [6,7]: A) age ≥ 17 years old; B) positive CSF culture in patients with clinical presentations of acute bacterial meningitis including fever, headache, altered consciousness and seizure; and C) at least one of the following CSF parameters: 1) a leukocyte count > 0.25 × 10^9^/L with predominant polymorphonuclear cells; 2) a CSF lactate concentration > 3.5 mmol/L; 3) a glucose ratio (CSF glucose/serum glucose) < 0.4 or CSF glucose concentration < 2.5 mmol/L if no simultaneous blood glucose was determined. The hospital's Ethics Committee approved the study (IRB 99-1897C).

"Nosocomial" meningitis was defined as a positive bacterial infection not present when the patient was admitted to the hospital, clinical evidence of an infection no sooner than 48 hours after admission, or clinical evidence of meningitis within one month after discharge from the hospital where the patient had received an invasive neurosurgical procedure. Otherwise the patient was considered to have "community-acquired" meningitis. Meningitis related to traumatic skull fracture, neurosurgical procedure or any causes of skull defects was classified as "post-neurosurgical" form. Otherwise, patients were classified as the "spontaneous" form. Patients aged ≥ 65 years were classified in elderly group, whereas patients' age < 65 years and ≥ 17 years were classified in non-elderly group [2,8]. "Mixed-infection" was defined as at least two bacterial organisms isolated from CSF culture [7,9].

The analysis of antibiotic susceptibility was based on the American National Committee for Clinical Laboratory Standards (NCCLS) or Clinical and Laboratory Standards Institute (CLSI) standard methods. In this study period, vancomycin plus a 3^rd^-or 4^th ^generation cephalosporin were the initial empiric antibiotics used in the treatment of patients with suspected ABM in our hospital, and the antimicrobial regimen was adjusted subsequently after the culture results were available.

For statistical analysis, clinical characteristics and therapeutic outcome between the patient groups (elderly group and non-elderly group) were compared. In the meanwhile, the clinical characteristics between the fatal and non-fatal cases of the elderly ABM patients were also compared. Data including gender, underlying condition, clinical manifestations, and therapeutic outcome were analyzed by means of Fisher's exact test. Age given for the two patient groups (elderly group vs. non-elderly group, fatal group vs. non-fatal group of the elderly ABM patients) was compared using the Mann-Whitney U test. Logistic regression of independent factor analysis was also performed. A *p*-value of < 0.05 was considered to be statistically significant.

## Results

**Figure 1:**
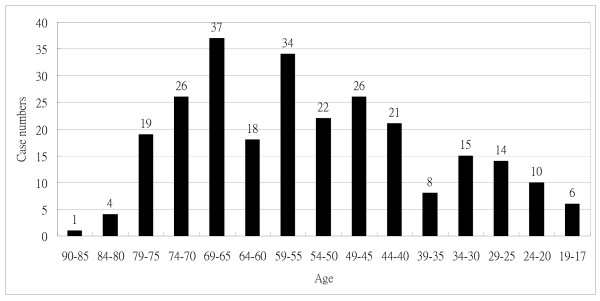
**Age distribution of the 261 patients with adult bacterial meningitis**.

During the study period, 261 patients with ABM were identified, and of them, 221 were found to have monomicrobial infection while the other 40, mixed infection. The age distribution of these 261 ABM patients is shown in Figure 1. Of the 261 cases with ABM, 87 belonged to the elderly group and the other 174, the non-elderly group. The implicated pathogens of the 261 patients with ABM are listed in Table 1. Seventy-seven of the 87 elderly ABM patients had monomicrobial infection and the other 10, mixed infection. The leading pathogens of monomicrobial G(+) and G(-) pathogen infection were staphylococcal species (17 cases) and *Klebsiella *(*K*.) *pneumoniae *(16), respectively.

**Table 1:** Implicated pathogens of the 261 elderly and non-elderly patients with bacterial meningitis

	Elderly patients	Non-elderly patients
	(n = 87)	(n = 174)
Gram positive	(n = 34) (39%)	(n = 62) (36%)
*Staphylococcus*	(n = 17) (19%)	(n = 38) (22%)
*Staphylococcus aureus *	11 (13%)	20 (11%)
*Coagulase- negative staphylococci*	1 (1%)	10 (6%)
*Staphylococcus epidermidis *	4 (5%)	6 (3%)
*Staphylococcus haemolyticus *		2 (1%)
*Staphylococcus saprophyticus*	1 (1%)	
*Streptococcus*	(n = 9) (10%)	(n = 16) (9%)
*Viridian streptococci*	3 (3%)	7 (4%)
*Streptococcus pneumoniae*	4 (5%)	8 (5%)
Group A beta-streptococci	1 (1%)	
Group B beta-streptococci	1 (1%)	1 (0.5%)
*Enterococcus *	(n = 6) (7%)	(n = 3) (2%)
*Enterococcus faecalis*	4 (5%)	3 (2%)
*Enterococcus facium*	1 (1%)	
*Enterococcus sp*	1 (1%)	
*Listeria monocytogenes*	1 (1%)	2 (1%)
*Corynebacterium*	1 (1%)	2 (1%)
*Micrococcus*		1 (0.5%)

Gram negative	(n = 43) (49%)	(n = 82) (47%)
*Acinetobacter*	(n = 9) (10%)	(n = 13) (7%)
*Acinetobacter baumannii*	7 (8%)	12 (7%)
*Acinetobacter lwoffii*	2 (2%)	
*Acinetobacter sp*.		1 (0.5%)
*Pseudomonas*	(n = 4) (4%)	(n = 13) (7%)
*Pseudomonas aeruginosa*	2 (2%)	9 (5%)
*Pseudomonas mendocina*	1 (1%)	1 (0.5%)
*Pseudomonas stutzeri*	1 (1%)	1 (0.5%)
*Pseudomonas putida*		1 (0.5%)
*Pseudomonas spp*.		1 (0.5%)
*Enterobacter*	(n = 4) (4%)	(n = 7) (4%)
*Enterobacter cloacae*	3 (3%)	5 (3%)
*Enterobacter aerogenes*	1 (1%)	2 (1%)
*Klebsiella pneumoniae *	16 (18%)	31 (18%)
*E coli*	4 (5%)	7 (4%)
*Salmonella *	2 (2%)	1 (0.5%)
*Proteus mirabilis*	2 (2%)	
*Serratia marcences*	1 (1%)	2 (1%)
*Elizabethkingia meningoseptica*	1 (1%)	
Others		8^a ^(4%)

Mixed infection	10 (11%)	30 (17%)

^a ^*Neisseria meningitidis *(2 cases), *Citrobacter diversus *(1), *Sphingomonas paucimobilis *(1), *Stenotrophomonas maltophilia *(1), unclassified glucose non fermenting group (2), and *Fusobacterium nucleatum *(1)

The clinical characteristics of the 87 elderly and the other 174 non-elderly ABM patients are listed in Table 2. The former 87 patients were 53 men and 34 women, aged from 65-87 years (median = 71 years). Of the underlying conditions, diabetes mellitus (DM) was the most common, found in 30 patients; followed by end stage renal disease, 6 patients; alcoholism, 4 patients and malignancies, 4 patients. Two of the latter 4 patients had lung cancer, and the other two had prostate cancer. As to the clinical manifestations of the 87 elderly ABM patients, fever was the most common, being found in 75 patients; followed by altered consciousness, 54 patients; leukocytosis, 46 patients; hydrocephalus, 33 patients; seizure, 26 patients; bacteremia, 18 patients, and shock, 10 patients. Thirty-nine of these 87 elderly ABM patients had spontaneous ABM, while the other 48, post-neurosurgical ABM. The duration between the last neurosurgical procedures and the development of bacterial meningitis ranged from 2 to 415 days (median 12 days) in the 48 elderly ABM patients. Forty-four patients contracted the ABM in a community-acquired state, while the other 43, a nosocomial state. The therapeutic result of the 87 elderly ABM patients was that 34 patients expired and 53 patients survived.

**Table 2:** Clinical comparison of the elderly and non-elderly patients with bacterial meningitis

**Factors**	**Elderly patients**	**Non-elderly patients**	***p***
	**(n = 87)**	**(n = 174)**	
	
Age (years); median (range)	71 (65 - 87)	48 (18 - 64)	
Gender			
Male	53	128	0.046*
Female	34	46	
Underlying condition			
Diabetes mellitus	30	38	0.036*
Liver cirrhosis	3	13	0.277
Alcoholism	4	12	0.590
End stage renal diseases	6	3	0.064
Malignancy	4	24^a^	0.032*
Spontaneous	39	52	0.019*
Community-acquired	44	67	0.084
Clinical presentation			
Fever	75	142	0.386
Altered consciousness	54	86	0.065
Seizure	26	41	0.294
Shock	10	17	0.671
Hydrocephalus	33	66	1.000
Brain abscess	7	18	0.659
Liver abscess	2	7	0.722
Positive blood culture	18	44	0.444
Leukocytosis	46	117	0.030*
Cerebrospinal fluid, median (IQR)			
White cell count (10^9^/L)	0.32 (0.07, 0.87)	0.37 (0.08, 2.00)	
Glucose (mmol/L)	2.50 (0.77, 4.20)	2.53 (0.33, 4.13)	
Protein (g/L)	1.85 (0.88, 4.11)	1.93 (0.68, 5.39)	
Lactate (mmol/L)	8.25 (4.29, 15.51)	8.31 (4.41, 16.15)	
Prognosis			
Survived	53	126	0.067
Expired	34	48	

*Fisher's exact test (p < 0.05); **Mann-Whitney U test (p < 0.05)

Logistic regression analysis showed independent factor of "leukocytosis" (p = 0.018)

IQR = inter-quartile range (25%, 75%)

^a ^Nasopharyngeal carcinoma (NPC) in 10 patients, lung cancer in 6 patients, breast cancer in 3 patients, hepatocellular carcinoma (HCC) in 2 patients, Hodgkin's lymphoma in 1 patient, plasma cell leukemia in 1 patient, buccal cancer in 1 patient, bladder in 1 patient and both NPC and HCC in 1 patient.

Table 2 shows the comparative results of the clinical and laboratory characteristics between the elderly and non-elderly ABM groups. Gender, presence of DM and malignancy, spontaneous infection and peripheral blood leukocytosis were significant factors. After logistic regression analysis, only peripheral blood leukocytosis was significant. Table 3 shows the comparative results of the clinical and laboratory characters between the fatal and non-fatal patients in the elderly group. The presence of end stage renal disease, spontaneous infection, seizure, shock and bacteremia were significant factors. After logistic regression analysis, only shock (*p*= 0.003) and seizure (*p *= 0.002) were significant.

**Table 3:** Prognostic factors of the 87 elderly patients with bacterial meningitis

	**Survived**	**Expired**	***p***
	**(n = 53)**	**(n = 34)**	
	
Age (years); median (range)	71 (65 - 82)	75.1 (66 - 87)	0.659
Gender			
Male	32	21	1.000
Female	21	13	
Underlying condition			
Diabetes mellitus	20	10	0.493
Liver cirrhosis	1	2	0.558
Alcoholism	1	3	0.295
End stage renal disease	0	6	0.003*
Malignancy	2	2	0.642
Spontaneous	19	20	0.047*
Community-acquired	25	19	0.512
Clinical presentation			
Fever	46	29	1.000
Altered consciousness	31	23	0.498
Seizure	11	15	0.030*
Shock	1	9	0.001*
Hydrocephalus	20	13	1.000
Brain abscess	4	3	1.000
Liver abscess	2	0	0.518
Positive blood culture	6	12	0.013*
Leukocytosis	25	21	0.196
Cerebrospinal fluid, median (IQR)			
White cell count (10^9^/L)	0.18 (0.03, 0.83)	0.45 (0.12, 1.27)	
Glucose (mmol/L)	3.16 (1.29, 4.32)	1.98 (0.48, 4.00)	
Protein (g/L)	1.09 (0.74, 3.32)	2.72 (1.58, 5.31)	
Lactate (mmol/L)	4.01 (3.16, 14.68)	9.17 (5.35, 17.13)	

* Fisher's exact test (p < 0.05); ** Mann-Whitney U test (p < 0.05)

IQR = inter-quartile range (25%, 75%)

## Discussion

In Taiwan, as data from the Taiwan government's Department of Health (Year 2009) has shown, the elderly accounted for 10.63% of the total (23,119,772) Taiwanese population whereas the age group of 15-64 years accounted for 73.03% [10]. These figures of age group distribution may mean that elderly persons accounted for 12.71% (10.63/83.66) of the group of persons with an age of ≥ 15 years. In the present study, we may find that the elderly group accounted for 34.8% (87/261) of the overall ABM patients with an age ≥ 17 years, and this figure of incidence is much higher than that of similar reports on bacterial meningitis in the US (1998-2007) which showed a 20% incidence of elderly patients among the overall ABM patients [6]. This figure of incidence may also confirm the belief that the elderly adults are more vulnerable to infectious diseases including bacterial meningitis than non-elderly adults. The increasingly aged population in Taiwan [10] may also indicate that the burden of bacterial meningitis will increase gradually in the elderly population in Taiwan.

As to the implicated pathogens of the 87 elderly ABM patients, 88.5% (77/87) of them belonged to monomicrobial infection, while the other 11.5% (10/87), mixed infection. As to the implicated pathogens of the monomicrobial infection, G(-) pathogens accounted for 55.8% (43/77) of them, while the other 44.2%, G(+) pathogens. This distribution pattern of implicated pathogens was similar to that of non-elderly ABM cases, in which G(-) pathogen accounted for 56.9% (43/77) of them, while the other 43.1%, G(+) pathogens. Despite the fact that there were minor differences in the implicated G(-) and G(+) pathogens of the elderly and non-elderly AMB with monomicrobial infection, *K. pneumoniae *and staphylococcal species were the most common in the implicated G(-) and G(+) pathogens, respectively, of both elderly and non-elderly groups of ABM patients. These relative frequencies were also similar to the reported implicated pathogens of overall ABM in Taiwan [7], but were different to those reported in other studies of elderly ABM [2-4], in which *Streptococcus pneumoniae*, *Neisseria meningitidis *and *Listeria monocytogenes *were common pathogens. This difference in implicated pathogens may reflect the believing that several factors including geographic distribution may influence the epidemiologic trend of ABM [6,7].

As shown in Table 2 several clinical and laboratory factors were different between the elderly and non-elderly ABM patients; but among them, only a lower incidence of peripheral blood leukocytosis was of statistical significance. This phenomenon of immune senescence reflected in the finding of blunt leukocytosis response in elderly patients [11-14]. Although several other factors did not reach a statistical significance, the relatively higher incidence of female patients in the elderly group can be attributed to the relatively longer life expectancy in females in Taiwan [8]. NPC was the most common malignancy among the 24 non-elderly ABM cases with malignancy. The relatively high incidence of NPC in the patients aged 40 to 50 years and its association with G(-) bacterial meningitis in Taiwan has been previously studied [15-17]. DM is an important preceding factor of ABM in Taiwan, especially in those with *K. pneumoniae *infection [18]. The relatively higher incidence of DM in the elderly ABM group can be attributed to the fact that the prevalence of DM increases with age [19].

It is known that, in acute bacterial meningitis, old-age is a grave prognostic factor [5-7], and as shown in this study, the elderly ABM patients had a relatively higher mortality rate (43%, 34/87) than non-elderly ABM patients (26%, 48/147), although this age difference did not show a significant influence on the mortality rate. Several factors are known to have an influence on the therapeutic result of ABM [5-7]. In present study, only the presence of seizure and shock were of significant influence. The important influence of the presence of seizure and shock on the prognosis of ABM is also noted in other studies of bacterial meningitis [2,7,20].

There are several limitations of this study that are worth noting. First, because of the defined criteria used to include studied cases, some of the patients with atypical clinical and laboratory presentations would be missed, e.g. patients without culture-proved bacterial meningitis were not included. Second, this is a retrospective study; several factors, such as nutritional status, were not recorded in charts; therefore, they were not included for clinical and prognostic analysis. Third, no data from nation-wide study are available and therefore the real estimated incidence of ABM among the elderly individuals cannot be estimated accurately. And fourth, the long study period may integrate different issues of the global epidemiology because of modification of surgical procedures, antibiotic prophylaxis and antimicrobial resistance.

## Conclusion

In this study, elderly ABM patients account for 34.8% of the overall ABM patients, and these figures with regards to incidence are higher than that of western countries. Thus, this relatively high incidence may signify the future burden of ABM in the elderly population in Taiwan. The relative frequency of implicated pathogens of elderly ABM is similar to that of non-elderly ABM. Compared with non-elderly ABM cases, the elderly cases have a significantly lower incidence pf peripheral blood leukocytosis. The mortality rate of elderly ABM is high and the presence of shock and seizures are important prognostic factors.

## Competing interests

The authors declare that they have no competing interests.

## Authors' contributions

All authors have read and approved the submitted manuscript.

WAL and SFC contributed to the conception and design, data acquisition and analysis, and drafting and revision of the manuscript. NWT, CCC, WNC, CHL and CCC contributed to the conception and design, and clinical data analysis and CRH contributed to the conception and design, data analysis, and critical revision and final approval of the manuscript.

## Pre-publication history

The pre-publication history for this paper can be accessed here:

http://www.biomedcentral.com/1471-2318/11/91/prepub
